# Impact of alveolar corticotomy-assisted orthodontics on root resorption and treatment duration

**DOI:** 10.6026/973206300210058

**Published:** 2025-01-31

**Authors:** Mohammad Khursheed Alam, Mohammad Younis Hajeer, Norah Noman Mohamed Alkwaykabe, Alrouh Abdulaziz Alruwaili, Ahmed Abdulrazaq Zaal Alfaleh

**Affiliations:** 1 Department of Preventive Dentistry, College of Dentistry, Jouf University, Sakaka 72345, Saudi Arabia; 2 Department of Dental Research Cell, Saveetha Institute of Medical and Technical Sciences, Saveetha Dental College and Hospitals, Chennai 600077, India; 3 Department of Public Health, Daffodil International University, Dhaka 1207, Bangladesh, South Asia; 4 Department of Orthodontics, University of Damascus, Damascus P.O. Box 16046, Syria

**Keywords:** Alveolar corticotomy, orthodontics, root resorption, treatment duration, accelerated orthodontics

## Abstract

The impact of alveolar corticotomy-assisted orthodontics (ACAO) on root resorption and treatment duration in orthodontic patients is of interest. Fifty patients were randomly divided into ACAO and control groups. Root resorption was assessed using CBCT and treatment duration recorded. The ACAO group exhibited significantly shorter treatment duration (10.2 ± 1.8 months) compared to the control group (16.5 ± 2.1 months, p<0.05). A slight increase in root resorption was observed in the ACAO group (0.8 ± 0.3 mm) versus the control (0.5 ± 0.2 mm, p<0.05), but no severe resorption was detected. ACAO effectively accelerates orthodontic treatment without clinically significant root resorption.

## Background:

Orthodontic treatment aims to achieve optimal dental alignment, occlusal function and facial esthetics. However, one of the most common concerns associated with traditional orthodontic therapy is its prolonged duration, which can impact patient compliance and increase the risk of adverse effects, such as root resorption, periodontal complications and enamel demineralization [1, 2]. To address these challenges, various surgical and non-surgical methods have been explored to accelerate tooth movement, with alveolar corticotomy emerging as one of the most effective approaches [3]. Alveolar corticotomy-assisted orthodontics (ACAO) is a surgical technique that involves selective cortical bone incisions to enhance tooth movement by inducing a transient osteopenic state known as the Regional Acceleratory Phenomenon (RAP) [4]. The RAP mechanism facilitates bone remodeling, allowing teeth to move more efficiently within the alveolar bone [5]. Compared to conventional orthodontic treatment, ACAO not only shortens treatment duration but also offers potential benefits such as reduced risk of root resorption and better periodontal outcomes when performed correctly [6]. Despite these advantages, concerns regarding the potential for increased root resorption in ACAO remain. Root resorption is a multifactorial process influenced by treatment mechanics, patient-related factors and the biological response of dental tissues to orthodontic forces [7, 8]. While some studies report that ACAO does not significantly increase the risk of root resorption compared to conventional orthodontics, others suggest the need for further evaluation, particularly with advanced imaging techniques such as cone-beam computed tomography (CBCT) [9, 10]. Therefore, it is of interest to evaluate the impact of ACAO on root resorption and treatment duration in patients undergoing orthodontic treatment.

## Materials and Methods:

This prospective study was conducted to evaluate the impact of alveolar corticotomy-assisted orthodontics (ACAO) on root resorption and treatment duration in patients undergoing orthodontic therapy.

## Study design and participants:

Fifty patients aged 18-30 years, requiring fixed orthodontic treatment, were recruited based on the following inclusion criteria: absence of systemic diseases, no history of periodontal disease and no previous orthodontic treatment. Exclusion criteria included smoking, pregnancy, or conditions affecting bone metabolism. Participants were randomly assigned into two groups: the ACAO group (n=25) and the control group (n=25). Randomization was achieved using a computer-generated random number sequence to ensure unbiased allocation.

## Alveolar corticotomy procedure:

The ACAO group underwent surgical corticotomy prior to the initiation of orthodontic treatment. The procedure was performed under local anesthesia using piezosurgical instruments to create precise cortical bone incisions in the alveolar bone. A full-thickness mucoperiosteal flap was raised to expose the alveolar bone and vertical and horizontal cuts were made to facilitate bone remodeling. Care was taken to avoid injury to the roots of the teeth. Following the procedure, the flap was repositioned and sutured and the patients were prescribed antibiotics and analgesics to manage postoperative discomfort.

## Orthodontic treatment protocol:

Both groups received standard fixed orthodontic treatment using 0.022-inch slot pre-adjusted edgewise appliances. Light continuous forces were applied using nickel-titanium archwires and adjustments were made at four-week intervals. In the ACAO group, orthodontic activation began one week after surgery to capitalize on the regional acceleratory phenomenon. The control group followed a conventional treatment timeline without surgical intervention.

## Assessment of root resorption:

Root resorption was assessed using cone-beam computed tomography (CBCT) imaging at two time points: baseline (before treatment) and at the end of orthodontic therapy. CBCT scans were obtained using standardized settings (85 kV, 7 mA and 10 seconds) and analyzed by two independent examiners blinded to the group assignments. Measurements were made for each tooth to determine the extent of apical root resorption, with differences in pre- and post-treatment lengths recorded in millimeters.

## Evaluation of treatment duration:

The total treatment duration, defined as the time from the placement of the first orthodontic archwire to the removal of the fixed appliance, was recorded for all participants. Treatment progress was closely monitored to ensure adherence to the planned protocols and to identify any complications.

## Statistical analysis:

Data were analyzed using statistical software (SPSS version 26.0). Descriptive statistics were used to summarize baseline characteristics, root resorption measurements and treatment duration. Independent t-tests were performed to compare the mean root resorption and treatment duration between the ACAO and control groups. A p-value of less than 0.05 was considered statistically significant.

## Results:

## Treatment duration:

A significant reduction in treatment duration was observed in the alveolar corticotomy-assisted orthodontics (ACAO) group compared to the control group. The mean treatment duration in the ACAO group was 10.5 ± 1.7 months, while it was 16.3 ± 2.2 months in the control group, with a statistically significant difference (p < 0.05). Detailed values are provided in Table 1.

## Root resorption:

The extent of root resorption was slightly higher in the ACAO group compared to the control group. In the ACAO group, the mean root resorption was 0.8 ± 0.3 mm, while it was 0.5 ± 0.2 mm in the control group and this difference was statistically significant (p < 0.05). However, the resorption observed in both groups remained within clinically acceptable limits. The data are summarized in Table 2.

## Compliance and complications:

Both groups exhibited good compliance with the treatment protocols. Postoperative discomfort in the ACAO group was mild and resolved within one week. No severe complications, such as periodontal issues or excessive root resorption, were observed in either group. The results indicate that ACAO significantly reduces treatment duration (Table 1) while causing a slight but clinically insignificant increase in root resorption (Table 2). These findings suggest that ACAO is an effective and safe adjunct to conventional orthodontic therapy.

## Discussion:

The findings of this study demonstrate that alveolar corticotomy-assisted orthodontics (ACAO) significantly reduces treatment duration while causing only a slight increase in root resorption compared to conventional orthodontic therapy. These results align with previous research highlighting the efficacy of ACAO in accelerating orthodontic tooth movement [1, 2]. The reduction in treatment duration observed in the ACAO group is primarily attributed to the Regional Acceleratory Phenomenon [RAP], which enhances bone remodeling and facilitates faster tooth movement [3]. Several studies have corroborated this mechanism, reporting treatment time reductions ranging from 25% to 60% with corticotomy-assisted techniques [4, 5]. For instance, Wilcko *et al.* [6] reported significant acceleration in orthodontic treatment when combining corticotomy with bone grafting, emphasizing the effectiveness of RAP. Similarly, a study by Abbas *et al.* [7] highlighted that corticotomy-facilitated orthodontics and piezocision are effective alternatives for accelerating canine retraction while minimizing root resorption in adult patients. Root resorption, although slightly higher in the ACAO group, remained within clinically acceptable limits. This is consistent with findings from Harris *et al.* [8], who reported that the risk of severe root resorption in accelerated orthodontics is low when appropriate forces are applied. It is hypothesized that the transient osteopenic state induced by corticotomy may mitigate some of the mechanical stresses associated with orthodontic forces, reducing the likelihood of excessive resorption [9]. Nonetheless, care must be taken to avoid overloading teeth during accelerated treatment, as excessive forces have been shown to exacerbate root resorption [10]. The use of cone-beam computed tomography (CBCT) in this study provided accurate and reproducible measurements of root resorption. CBCT imaging has been widely endorsed as a reliable method for assessing orthodontic outcomes, including root morphology and bone changes [11, 12]. Study by Alamadi *et al.* [13] has emphasized the importance of CBCT in detecting subtle resorption changes that may not be apparent in conventional radiographs. Despite its benefits, ACAO is not without limitations. The surgical procedure, although minimally invasive, carries risks such as postoperative discomfort, swelling and the potential for infection. However, these complications can be minimized with proper surgical techniques and postoperative care [14]. Additionally, patient acceptance of the procedure may vary, necessitating thorough counseling to address concerns and set realistic expectations. This study has some limitations, including a relatively small sample size and short follow-up period. Long-term studies with larger cohorts are needed to assess the stability of treatment outcomes and the potential for relapse. Future research could also explore the integration of ACAO with other accelerated orthodontic techniques, such as micro-osteoperforations or vibration devices, to further enhance treatment efficiency.

## Conclusion:

Alveolar corticotomy-assisted orthodontics is a promising adjunctive technique that significantly reduces treatment duration with minimal risk of clinically significant root resorption. It can provide faster and predictable results while improving patient satisfaction and compliance when performed with careful planning and execution.

## Figures and Tables

**Table 1:** Comparison of treatment duration between groups

**Group**	**Mean Treatment Duration (months)**	**Standard Deviation**	**p-value**
ACAO Group	10.5	1.7	< 0.05
Control Group	16.3	2.2	

**Table 2:** Comparison of root resorption between groups

**Group**	**Mean Root Resorption (mm)**	**Standard Deviation**	**p-value**
ACAO Group	0.8	0.3	< 0.05
Control Group	0.5	0.2	
